# Upper Airways Spray for Viral Infections Prevention

**DOI:** 10.1155/2022/2502199

**Published:** 2022-10-04

**Authors:** Beniamino Palmieri, Maria Vadalà

**Affiliations:** ^1^Medico Cura Te Stesso Onlus, Modena, Italy; ^2^Second Opinion Medical Network, Modena, Italy

## Abstract

**Aim:**

Several studies emphasized the antiviral properties of many natural compounds enclosed in nutraceuticals formulas and quite effective to prevent the respiratory infections. The rationale of our investigation has been to achieve protection from common cold viruses' infection of the upper airways pooling together and dispensing different active principles on a multistep defense basis. *Material and Methods*. 30 patients affected by sudden aspecific viral-induced sore throat rhinitis were divided in two groups: (1) the first group included 15 patients which were administered with our spray formula and (2) the second group included 15 patients with the commercial nasal lavage kit. The mucous smear was stained with May Grunwald-Giemsa to exclude eosinophilic infiltrate and confirm the prevalence of granulocytes and lympho-monocytes typical of viral seasonal inflammatory upper airways conditions.

**Results:**

The symptomatic relieve is remarkedly evident in the treated group with our spray compared to the second group treated with commercial nasal lavage kit.

**Conclusions:**

The open case-control retrospective observational study showed a definite benefit of the spray based on natural herbal extracts to take control of the upper airways respiratory distress due to viral infections.

## 1. Introduction

Many viral pathogenic agents enclosing influential respiratory syncytial rhinovirus, herpes, and several other agents (such as SARS-CoV-2) harbor usually along the upper airways mucosae and spread the infection into the trachea, bronchus, and lungs and even the bowel with the possible renown clinical complications (Tables 1 and 2).

Furthermore, sometimes even in healthy carriers, the nasopharyngeal diagnostic swab can remain virus positive even for weeks or months after the complete resolution of the symptoms.

Several clinical studies faced in the last 3 years the prevention of virus entrance through the nostrils and the throat by means of nasal irrigation with antiseptics, drops, or sprays to inactivate the pathogenic agents. As to the different marketing available tools, the nasal sprays are based on antiseptics (chlorhexidine and iodine povidone), antihistaminic, and vasoconstrictors, while the oral ones being mainly based on generic antiseptics but also anti-inflammatory and natural compounds such as propolis.

In this perspective, the antiviral properties of many natural compounds have been investigated as effective nutraceuticals formulas to achieve preventive control of the respiratory infections.

Our proposal is based on a mix of herbal extracts such as *Phyllantus* and *Andrographis paniculata,* added with copper ions and zinc. The activity of each herbal strain can be summarized as follows:

1. *Phyllanthus* family involves *Niruri* and/or *Phyllantus Emblica, Amarus*, and *urinaria.,* leaves extract to concentration range among 2 and 15%. This extract is composed by secondary metabolites including flavonoids, tannins, lignins, polyphenols, and triterpenes, and displays several ethnopharmacological actions, e.g., anti-inflammatory, hepatoprotective, nephroprotective, anticancer, antioxidant, and antiviral, particularly in conditions including diarrhea, dysentery, dropsy, running nose, winter common colds [27, 28]. Yang et al. [29] enhanced the antiviral role of extracts of *Phyllanthus urinaria* against Herpes Simplex Virus 1 (HSV-1), strain KOS, and Herpes Simplex Virus 2 (HSV-2). In particular, the acetone (31.4%), ethanolic (40.4%), and methanolic (41.3%) extractants inhibited more than 90% of HSV-2 plaques. HSV-2 were destroyed when the compound was added to cells just after viral infection rather than added pre- or postviral infection
2. *Andrographis paniculata* is widely used in the eastern folk medicine for relieving and decreasing the typical symptoms of flu, such as fever, cough and sore throats, *in vitro*, it suppresses avian influenza A (H9N2 and H5N1) and human influenza A H1N1 viruses, maybe through blocking the binding of viral hemagglutinin to cells [30], or by inhibiting H1N1 virus-induced cell death [31]. *A. paniculata* extracts were analyzed against a wide variety of pathogens, such as several antibiotic-resistant species, e.g., *Staphylococcus aureus, Pseudomonas aeruginosa, Salmonella* spp.*, Candida* spp., and *Streptococcus pneumoniae*. Fifty-nine invasive microbes have been used to analyze the antimicrobial efficacy of *A. paniculata* extracts and/or their isolated pure compounds. Its active components named “andrographolides” include the diterpene, lactones which provided anti-inflammatory, antiviral, and immune-stimulatory activities [32]. They inhibit platelet-activating factor mediated inflammatory response [33], reduce expression of proinflammatory proteins (e.g., cyclooxygenase-2) [34, 35], and demonstrate analgesic effects, in addition to antipyretic effects comparable to paracetamol [36]
3. Copper (Cu) ions are very active against several viruses responsible of pneumonitis, enclosing Polio, and the SARS-CoV-2. Adhesion to the respiratory mucosae is enhanced by poloxamer or carboxymethyl cellulose. Cu modulates the morphology and action of neutrophils, NK cells, macrophages, T helper cells, and B cells, on killing infectious pathogens, activation of cell-mediated immunity, and secretion of specific antibodies. Neutropenia and lymphopenia have been studied in Cu deficient cases. Subsequently, insufficient Cu status leads to immunosuppression, rising susceptibility to infectious diseases [37]. A raised serum Cu content exists in response to inflammation, provoking Cu to accumulate at inflammatory sites. Cu may suppress the production of inflammatory cytokines, chemokines, and adhesion molecules by downregulating the expression of nuclear factor kappa-light-chain-enhancer of activated B cells (NF-*κ*B), which is generally activated by virus-induced ROS [38, 39]. Cu is a powerful virucidal element, on many infectious viruses, such as the bronchitis virus, influenza virus, HIV type 1, and other, single- or double-stranded DNA and RNA viruses [40, 41]. During viral infection, Cu acts as an essential micronutrient for both pathogens and animal hosts but not at high toxic levels triggering undue redox reactions. This occurrence can be explained by the fact that macrophages can attack pathogens at high Cu load during an infection, but higher Cu levels have been found in the sites of lung infection [42, 43]. Reliably, Cu inhibits influenza virus replication by stopping the action of RNA-dependent RNA polymerase (RdRP) or damaging its negative-sense RNA [40]. Copper iodide induces the production of free radicals (-OH and -O_2_), which exhibit virucidal actions by the degradation of viral proteins, e.g., hemagglutinin and neuraminidase [44]. Cu meddles with main proteins of the virus. *In vitro* studies have reported that Cu action caused 99% inactivation of viruses after 30 minutes, and the effect was observed to be more obvious in enveloped viruses [45]. Oxidized Cu oxide (CuO) nanoparticles are utilized as catalysts that determine inactivation of viruses. Similarly, nano-sized Cu iodide particles have also been presented to display the inactivation of the H1N1 influenza virus. Cu exerts a stimulatory role on ceruloplasmin expression, a major copper-carrying protein in the blood that boosts the immune response during inflamed/infectious actions
4. Zinc (Zn) has many direct and indirect antiviral properties. Zn homeostasis is connected with infections related to coronaviridae [46], picornavirus [47], papilloma virus [48], rhinovirus [49], herpes simplex virus [50], varicella-zoster virus [51], respiratory syncytial virus (RSV) [52], human immunodeficiency virus (HIV) [53], and hepatitis C virus (HCV) [54]. Zn shows antiviral actions in physical phases, e.g., the infection and replication of virus [55, 56]. Zn is involved in the expression of different cellular enzymes and transcription factors being cofactor of many viral proteins as well as rendering easier and faster the polyproteins misfolding of viral proteins, which alters the architecture of the virus modulating its protease activity, as shown in the picorna and polioviruses [47, 57, 58]. An *in vitro* study displayed that Zn can inactivate the free varicella-zoster virus [51]. Zn salts facilitate viral destruction by abolishing viral entry, polyprotein processing, or viral RNA-dependent RNA polymerase (RdRP) activity in other viruses, such as SARS-CoV [46], rhinovirus [49], HSV [50], HIV [53], and vaccinia virus [59]. Zn behaves as a membrane stabilizer that may prevent the entry of the virus into the cell. [55]. The usage of Zn also reduces the oxidative stress induced by the RSV and influenza virus by triggering metallothioneins (MTs) to release Zn into the cytoplasm, which maintains the cellular redox state [60]. A significant reduction in the levels of plasma Zn levels in patients with acute respiratory distress syndrome (ARDS) suggests that Zn could be helpful in improving the clinical conditions of mechanically ventilated patients [61]
5. Poloxamer (Pluronic) is part of the family of hydrogels polymeric materials provided of a three-dimensional network that can retain a large amount of water or biological fluid under physiological conditions. They could be used as delivery systems due to the unique properties of sol–gel conversion that is modulated by a specific biological stimulus [62]. In our case, poloxamer 407 (P407) is used for its good solubilizing capacity, low toxicity, good drug-release characteristics, and its compatibility with several natural compounds and excipients [63]. P407 is useful mucosal drug delivery systems, due to its coating a thin layer on the biomembranes, thus delivering active principles along the compartment, at a long-term steady concentration. Poloxamer molecules produce entanglements or noncovalent bonds with mucus, thus intensively interacting with the epithelial background in a safe and effective barrier against noxious agents

Our strategy has been to share protection from common cold viruses' infection of the upper airways pooling together and dispensing different active principles on a multistep defense basis.

## 2. Materials and Methods

The study was done using 30 cases of infected patients by sudden aspecific viral-induced sore throat rhinitis excluding bacterial origin by preliminary microbiological swab ruling out infections of *Streptococcus*, *Staphylococcus aureus*, *Hemophilus*, etc., just limiting the diagnosis and patients' selection to the common cold viruses' group such as influential, rhinovirus, and adenovirus. The study is a simple open investigation comparing two groups of subjects admitted to the Second Opinion Medical Consulting Network at the first arousal of the symptoms and submitted to diagnostic nasal and oral swab. The Second Opinion Medical Consulting Network is a medical consultation referral web, quickly recruiting a wide panel of real-time available specialists, to whom any patient affected by any disease or syndrome not adequately satisfied by the first diagnosis or therapy, can apply for an individual clinical audit [64–68]. We usually after a careful review of the diagnosis plan for the patient's tailored proper therapeutic strategy with chemical or natural drugs or galenic formulations prepared by the pharmacists or nutraceutical manufacturing companies∗.

The patients were divided in two groups comparable in terms of age, clinical history, symptoms: (1) the first group included 15 patients, which were administered with our spray formula, and (2) the second group included 15 patients with a commercial nasal lavage kit (Table 3). The mucous smear was stained with May Grunwald-Giemsa to exclude eosinophilic infiltrate and confirm the prevalence of granulocytes and lympho-monocytes typical of viral seasonal inflammatory upper airways conditions.

They were then instructed to take daily record of the symptoms and to use the spray (our spray and commercial spray, respectively, in the first and second group) every 8 hours for 5 days, unless remission had been detected in advance.

Statistical analyses were performed using GraphPad Prism 7 (GraphPad Software Inc., San Diego, CA, USA). The data were examined using an unpaired *t*-test with Welch's correction. *p* < 0.05 was considered significant.

∗In this study, the nasal spray samples conventionally labeled “Trivir” were kindly prepared free of charge on our prescription by Phytoitalia (Milan, Italy).

## 3. Results

**Figure 1:**
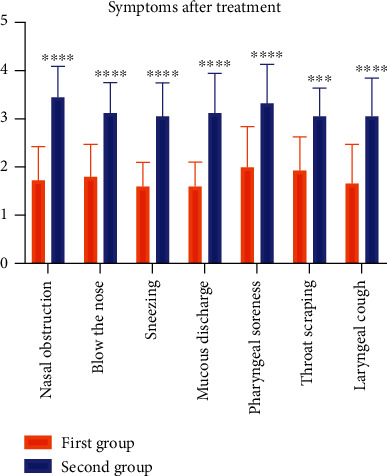
Graphical representation of symptoms in first and second group after treatment. There were significant differences. ∗∗∗∗*P* < 0.0001 first- vs. second group.

The results in Table 3 report the symptoms recorded by the first group of patients, treated with our spray. A significative reduction of the main influential symptoms was detected (Table 4, Figure 1).

The patients of the second group recovered more slowly and with persistent symptoms compared with the first group (Table 5, Figure 1).

Anecdotally, we showed in (Table 6) how further 10 healthy carriers of SARS-CoV-2 in the nostrils and upper airways become quickly negative using our spray and reporting the diagnostic swab every three days.

## 4. Discussion and Conclusions

The open case-control retrospective observational investigation showed a definite benefit of the spray based on natural herbal extracts to take control of the upper airways respiratory distress due to viral infections, as a matter of fact, the symptomatic relief is reported in Table 6. The results are remarkably evident in the treated group with the spray treatment compared to the control group, to whom oral medications antihistamines, vasoconstrictors, salicylates, or local antigrippal formulas had been prescribed.

This study is very basic in terms of design and concepts; however, the lesson that folk medicine taught us about natural products has been very helpful. In fact, the control of the seasonal cold symptoms, achieved in the present study with herbal extracts plus divalent cations, is worth of further investigation.

The main limitation of our study was the small cohort of patients, consequently we cannot exclude error rates (Type 1 and Type 2 errors) and cannot ensure that our results may be replicated in future research with a major patients group. But this preliminary observation and the positive outcomes are very promising and recommend further major evidence-based clinical trial.

Obviously, the pharmaceutical technology of adhesion molecules, namely, the poloxamer steadily and firmly coating the herbal active principles along the mucosal surfaces ideally integrates the antivirus action mechanism fulfilling the scope of the formula.

## Figures and Tables

**Table 1:** Common pathogens concerned in respiratory tract infections (bacteria, viruses, and fungi).

Pathogen	Type gram	Pathology	Ref.
*Streptococcus pneumoniae*	+	Bacterial pneumonia	[1, 2]
*Haemophilus influenzae*	−	Pneumonia, sinusitis	[3, 4]
*Chlamydophila pneumoniae*	Obligate intracellular bacterium	Atypical pneumonia	[5, 6]
*Staphylococcus aureus*	+	Sinusitis, pneumonia	[7, 8]
*Pseudomonas aeruginosa*	−	Sinusitis, pneumonia	[9]
*Legionella pneumophila*	−	Cough with sputum, bronchiolitis	[10, 11]
*Moraxella catarrhalis*	−	Bronchitis, sinusitis, and bronchopneumonia	[12]
Rhinoviruses	Enterovirus	Common cold and sinusitis	[13]
Coronaviruses	Coronavirinae	Pneumonia	[14, 15]
Respiratory syncytial virus	Pneumovirus	Bronchiolitis	[16]
Adenovirus	Adenoviridae	Pneumonia and tonsillitis,	[17]
Herpes simplex virus	Respirovirus	Pneumonia	[18, 19]
*Histoplasma capsulatum*	*Histoplasma* (dimorphic fungi)	Pneumonia	[20]
*Cryptococcus neoformans*	*Cryptococcus* (yeast)	Pneumonia	[21, 22]
*Coccidioides immitis*	*Coccidioides* (pathogenic fungus)	Pneumonia	[23, 24]
*Pneumocystis jirovecii*		Pneumonia	[25, 26]

**Table 2:** Estimated annual proportion of clinical case based on virus' type.

Virus's type	Estimated annual proportion of cases
Rhinovirus	30–50%
Coronavirus	10–15%
Influenza virus	5–15%
Respiratory syncytial virus	5%
Parainfluenza virus	5%
Adenovirus	<5%
Enterovirus	<5%
Metapneumovirus	Unknown
Unknown	20–30%

**Table 3:** Characteristic of patients.

First group				Second group		
	Patient ID	Age (years)	Sex	Patient ID	Age (years)	Sex
#1	P.E.	76	M	D.B.	36	F
#2	M.G.	64	M	M.C.	73	M
#3	F.W.	55	F	A.C.	62	M
#4	V.F.	61	F	S.E.	76	M
#5	B.C.	66	M	A.U.	56	F
#6	S.V.	78	F	B.A.	59	M
#7	C.A.	56	M	F.M.	68	M
#8	P.M.	51	F	M.C.	49	M
#9	C.M.	62	M	S.E.	61	M
#10	R.S.	38	M	V.K.	76	M
#11	B.M.	47	M	A.G.	64	F
#12	L.A.	61	F	L.P.	45	F
#13	E.R.	54	F	A.T.	51	F
#14	M.B.	66	F	D.E.	38	F
#15	A.A.	36	M	I.F.	53	F

**Table 4:** Description of symptoms in the first group (our spray) after treatment.

First group	Symptoms posttreatment						
	Nasal obstruction	Blow the nose	Sneezing	Mucous discharge	Pharyngeal soreness	Throat scraping	Laryngeal cough
P.E.	Rare	Intermittent	Absent	Absent	Absent	Rare	Absent
M.G.	Rare	Absent	Intermittent	Absent	Persistent	Intermittent	Absent
F.W.	Absent	Absent	Rare	Intermittent	Intermittent	Rare	Absent
V.F.	Absent	Absent	Rare	Persistent	Rare	Rare	Rare
B.C.	Rare	Rare	Absent	Absent	Intermittent	Absent	Intermittent
S.V.	Rare	Absent	Intermittent		Absent	Rare	Rare
C.A.	Absent	Intermittent	Absent	Persistent	Rare	Rare	Absent
P.M.	Absent	Rare	Rare	Intermittent	Rare	Absent	Intermittent
C.M.	Rare	Intermittent	Absent	Intermittent	Absent	Rare	Rare
R.S.	Absent	Absent	Rare	Intermittent	Persistent	Intermittent	Persistent
B.M.	Rare	Absent	Absent	Absent	Absent	Rare	Intermittent
L.A.	Intermittent	Rare	Intermittent	Absent	Rare	Rare	Absent
E.R.	Rare	Absent	Absent	Rare	Rare	Absent	Absent
M.B.	Absent	Intermittent	Rare	Intermittent	Intermittent	Absent	Absent
A.A.	Intermittent	Rare	Rare	Rare	Absent	Intermittent	Rare

**Table 5:** Description of symptoms in the second group (commercial spray) after treatment.

Second group	Symptoms post-treatment						
	Nasal obstruction	Blow the nose	Sneezing	Mucous discharge	Pharyngeal soreness	Throat scraping	Laryngeal cough
D.B.	Persistent	Intermittent	Absent	Absent	Persistent	Rare	Persistent
M.C.	Rare	Absent	Intermittent	Absent	Persistent	Intermittent	Absent
A.C.	Rare	Intermittent	Persistent	Rare	Intermittent	Rare	Absent
S.E.	Intermittent	Absent	Rare	Persistent	Persistent	Intermittent	Rare
A.U.	Absent	Rare	Persistent	Absent	Intermittent	Persistent	Intermittent
B.A.	Intermittent	Persistent	Intermittent	Absent	Intermittent	Rare	Persistent
F.M.	Persistent	Intermittent	Absent	Persistent	Rare	Rare	Absent
M.C.	Absent	Persistent	Rare	Intermittent	Rare	Persistent	Intermittent
S.E.	Persistent	Intermittent	Absent	Absent	Persistent	Rare	Persistent
V.K.	Absent	Absent	Intermittent	Absent	Persistent	Intermittent	Absent
A.G.	Persistent	Intermittent	Persistent	Rare	Intermittent	Rare	Absent
L.P.	Intermittent	Absent	Rare	Persistent	Persistent	Intermittent	Rare
A.T.	Rare	Rare	Persistent	Absent	Intermittent	Rare	Intermittent
D.E.	Intermittent	Persistent	Intermittent	Persistent	Intermittent	Rare	Persistent
I.F.	Persistent	Intermittent	Absent	Persistent	Rare	Rare	Absent

**Table 6:** Case report of 10 healthy voluntaries, carriers of SARS-CoV-2, treated with our spray.

Case report	Sex	Age	2 consecutive swab before		Day 3	Day 5
Patient ID			Swab1	Swab2	Swab3	Swab4
D.B.	F	36	+ +	+ +	Negative	Negative
M.C.	M	73	+ +	+ +	Negative	Negative
A.C.	M	62	+ +	+ +	Negative	Negative
S.E.	M	76	+ +	+ +	Negative	Negative
A.U.	F	56	+ +	+ +	Negative	Negative
B.A.	M	59	+ +	+ +	+ +	Negative
F.M.	M	68	+ +	+ +	+ +	Negative
M.C.	M	49	+ +	+ +	Negative	Negative
S.E.	M	61	+ +	+ +	Negative	Negative
V.K.	M	76	+ +	+ +	+ +	Negative
Positivity index			10/10	10/10	3/10	0/10

## Data Availability

The authors declare that data supporting the findings of this study are available within the article.
